# A probe into probe use: decontamination challenges of point of care ultrasound (POCUS)

**DOI:** 10.1017/ash.2025.111

**Published:** 2025-09-03

**Authors:** Jincy Jerry, Vasantha Gopalan

**Affiliations:** Affiliation, Infection Prevention and Control DepartmentMater Misericordiae University Hospital, Dublin, Ireland

## Abstract

**Background:** Technological advancements have made ultrasound devices more portable and user-friendly. Even when ultrasound machines and probes are visibly clean, clinically important pathogenic organisms have been grown from them (Shokoohi et al, 2015). Ultrasound machines could therefore serve as a fomite for pathogens known to cause healthcare-associated infections. Thorough cleaning and disinfection of ultrasound equipment greatly reduces the microbial burden and lessens the chance of clinically important infections. However, studies indicate suboptimal cleaning practices and a lack of training among ultrasonographers [Westerway et al 2019]. **Aims and Objectives:** To assess the standard of ultrasound/probe cleaning within the hospital at POCUS and to benchmark it against hospital policy. Assess the level of probe reprocessing training received by operators. Develop and implement interventions based on targeted need assessment and evaluate their effectiveness. **Method and Outcome:** A study of ultrasound/probe reprocessing practices identified serious concerns. The cleaning of USG probes was suboptimal for critical, semi-critical, and non-critical probes as per the Spaulding classification. The compliance level for tracking and tracing was unacceptable. Lack of knowledge, inadequate access to cleaning supplies and equipment, and time constraints were primary barriers to guideline-based disinfection. Interventions were guided by the audit results. To better educate Ultrasonographers, an educational tool was created with best practices for USG machine and probe cleaning and disinfection, an instructional video, a summary of cleaning steps, and links to best-practice guidelines. We were able to significantly improve the thoroughness of cleaning ultrasound machines and probes by using targeted interventions. **Conclusion:** For ultrasound-guided procedures, non-compliance implies greater risks. The results of this study confirm the concern expressed by a global survey of ultrasound users which suggested that ultrasound cleaning procedures are inadequate and that users are unaware of recommended practices.

